# Methodology for hydraulic characterisation of the sand filter backwashing processes used in micro irrigation

**DOI:** 10.1016/j.mex.2020.100962

**Published:** 2020-06-13

**Authors:** Fábio Ponciano de Deus, Marcio Mesquita, Roberto Testezlaf, Rodrigo Cesar de Almeida, Henrique Fonseca Elias de Oliveira

**Affiliations:** a Federal University of Lavras, Water Resources and Sanitation Department, postal code 3037 Lavras, MG, Brazil; b Federal University of Goiás, Goiânia, GO, Brazil; c State University of Campinas, Campinas, SP, Brazil; d Federal University of Lavras, Lavras, MG, Brazil; e Federal Institute Goiano, Ceres, GO, Brazil

**Keywords:** Water treatment, Emitter clogging, Surface velocity, Fluidisation

## Abstract

The correct operation of the backwashing process in sand filters used in micro irrigation determines directly the effectiveness of the subsequent filtration processes, and consequently the micro irrigation systems maintenance. As backwashing involves the filter bed expansion, the current sand filter design makes it impossible to measure the process. In this sense, this work aims to present a hydraulic characterization methodology of the backwashing process of sand filters used in micro irrigation, evaluating the influence of the sand particle size, the filter bed height, and the sand filter design. The proposed methodology can assist manufacturers in gathering equipment operating information, aiming at its presentation in the product catalogs.

• This method article discusses a method of hydraulic evaluation of the backwashing process of sand filters used in micro irrigation.
• The method can assist sand filter manufacturers in the correct definition of the equipment's operating parameters.
• The method shows the effectiveness of using the filter media pressure loss evaluation in the indirect definition of the minimum fluidisation velocity.

## Grafical abstract

**Figure:**
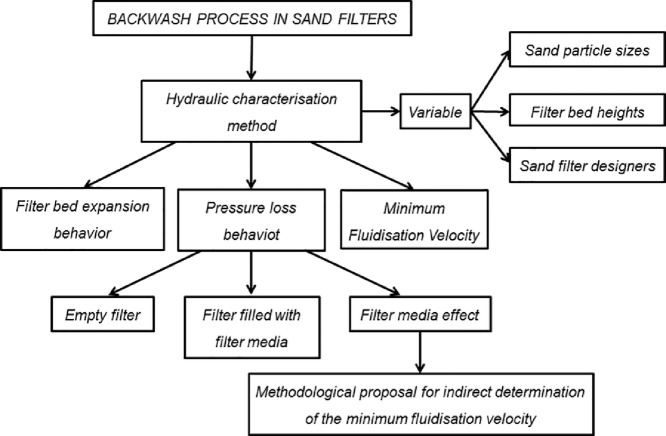

**Table:** **Specifications Table**

Subject Area:	Agricultural and Biological Sciences
More specific subject area:	Irrigation
Method name:	Sand filter backwash assessment method
Name and reference of original method:	N/A
Resource availability:	N/A

## Method details

**Fig. 1:**
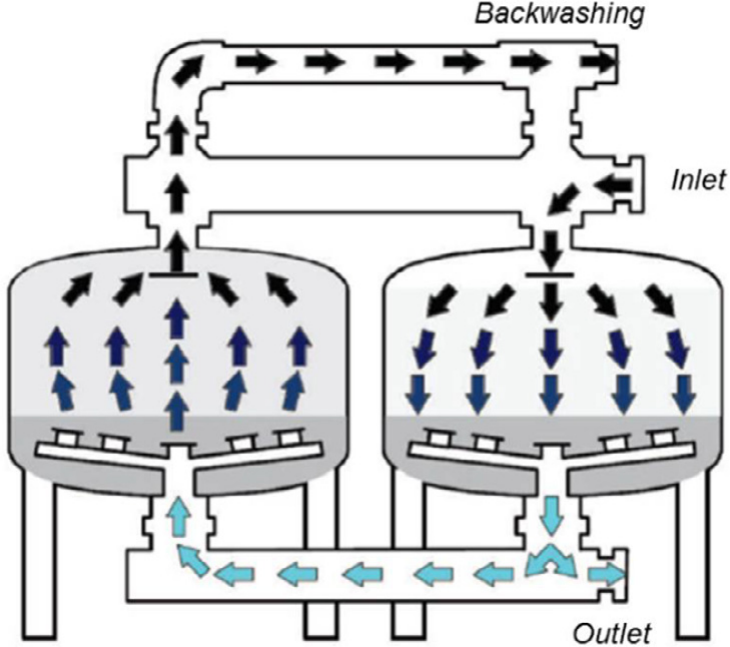
Backwash process in sand filters used in micro irrigation (adapted from the Flow-Guard (2013) [6]).

This article is a detail of the hydraulic evaluation methodology of the backwashing process of sand filters used in micro irrigation, of the article published by de Deus et al. (2020) [7]. Backwashing is the process of cleaning sand filters, with direction of the flow is opposite to that of filtration, i.e., upward [1,2]. In this process, the water previously filtered by one or more filters expands and disturbs the filter bed, and because there is a specific mass difference between the impurities and the filter media, the less dense particles, the impurities, are removed from the equipment [2,3]. This procedure promotes the filter bed expansion, which is a function of the surface velocity (flow rate ratio for the cross-sectional area of the sand filter), that according to Gupta and Sathiyamoorthy (1999) [4], this process involves a mechanism associated with fluidisation theory, that is a phenomenon of providing fluid properties to a solid particles layer, and determining its expansion. Knowing this process in detail is important to ensure the correct equipment operation [5], as several variables interfere, such as the sand particle size, the filter bed height, and the sand filter designs. Fig. 1 illustrates the backwash process in sand filters used in micro irrigation.

**Fig 2:**
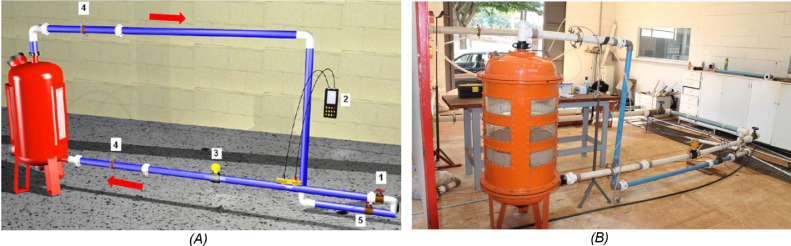
Diagram of the experimental module assembly with sand filters in a closed flow circuit, detailing the position of the equipment used in the experiment and the flow direction (1 and 5 – needle valves inlet and outlet respectively, 2 – ultrasonic flow rate, 3 – electromagnetic flow rate, and 4 – integral pressure sensors) (A), and a photo of the experimental module assembly (B).

**Fig 3:**
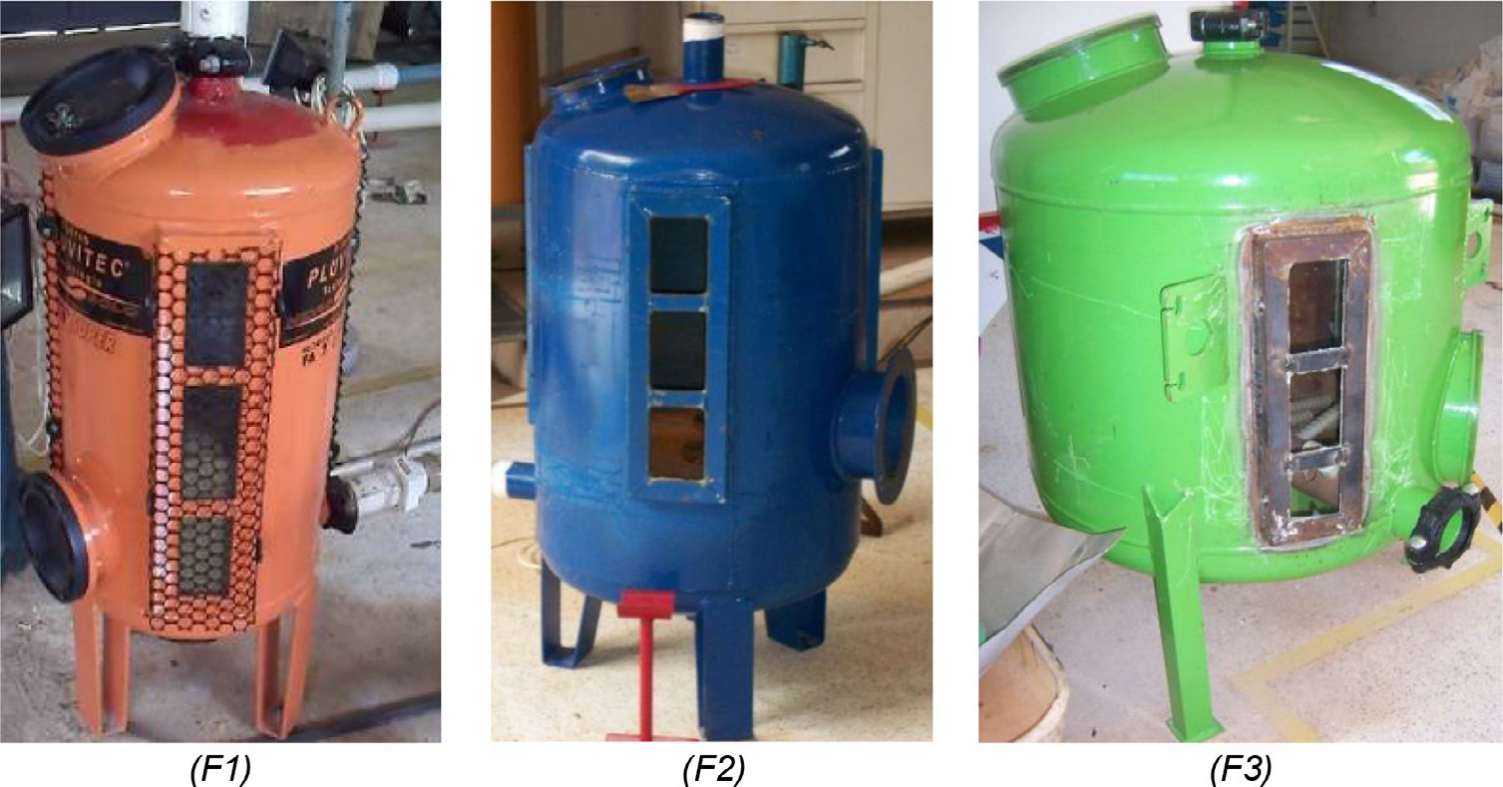
Commercial sand filters models evaluated, with detail of the tempered glass side panels installed.

The survey whose results were presented by de Deus et al. (2020) [7] proposed the construction of an experimental module in a closed circuit of water circulation for sand filter backwash assessments, composed of the following equipment (Fig. 2):

- Sand filter: The evaluated sand filters were from three Brazilian commercial brands, designated F1 (Hidro Solo), F2 (Marbella) and F3 (Amanco). The commercial sand filters used in micro irrigation are made of steel sheet, not allowing the visualization of the filter bed expansion. In order to evaluate the filter bed expansion in the backwashing processes, side viewers must be adapted to allow visualization. Fig. 3 shows the commercial sand filters evaluated in the research, with details of the tempered glass side panels installed.

For the structural characterization of sand filters, it is recommended that the following technical specifications are informed by the manufacturers: Diameter of the filter; Filtration surface; Internal diameter of the inlet pipe; Internal diameter of the outlet pipe; Useful height of the filter (Distance between the base of the diffuser plate and the upper level of the drains); Number of drains; Average width of a drain opening; Number of openings per drain; Opening area per drain unit; Total opening area of the drainage system; and Ratio between drainage area and filter area.

- Needle valves: the purpose of using this type of valve was to submit greater precision to the evaluations for the effective maintenance of the flow in time. Two valves must be used, one before and one after the evaluated sand filter.
- Flow rate sensor: to record the flow information over time it was used an eletromagnetic flow rate sensor (Signet 2551, of the Georg Fischer brand, Piping Systems Ltd., São Paulo, Brazil), and to instantly measure the flow it was used an ultrasonic flow rate sensor (UFM170, FMS, São Paulo, Brazil). The location of the sensors followed recommendations of the standard ASABE (1993) [8]. The standard establishes that the flow sensors must have a minimum precision of ± 2% in the evaluated flow range, and must be installed respecting a minimum length of 50 times the pipe internal diameter after the valve in the flow direction, and a minimum of five times the pipe internal diameter after the flow rate sensor. Table 1 shows the technical specifications of the flow rate sensors used.

**Table 1:** Technical specifications of the flow rate sensors used in the experiment.

Specification	Eletromagnetic flow rate	Ultrasonic flow rate
Pipe diameter range (mm)	12.7–304.8	12.7–7620
Flow range (m s^−1^)	0.05–10	−16 to 16
Accuracy (%)	0.5	0.5
Temperature range (°C)	−10 to 70	–
Maximum pressure a 25 °C (kPa)	1030	–
- Pressure transducers and integral pressure sensors: to measure the pressure loss was used two pressure transducers model MPX5700DP (Freescale Semi-conductor Brazil, Campinas, São Paulo, Brazil) (Fig. 4A), connected each to one integral pressure sensors that could measure pressure without producing pressure loss by insertion into the flow (Figura 4B). Table 2 shows the technical specifications of the pressure transducers model used in experiment. The pressure information comes from converting the voltage generated by the equipment. The pressure transducer converts the pressure (P) in the pipe into voltage (V_out_), and using a transfer function it is possible to estimate the pressure value (P).

**Fig 4:**
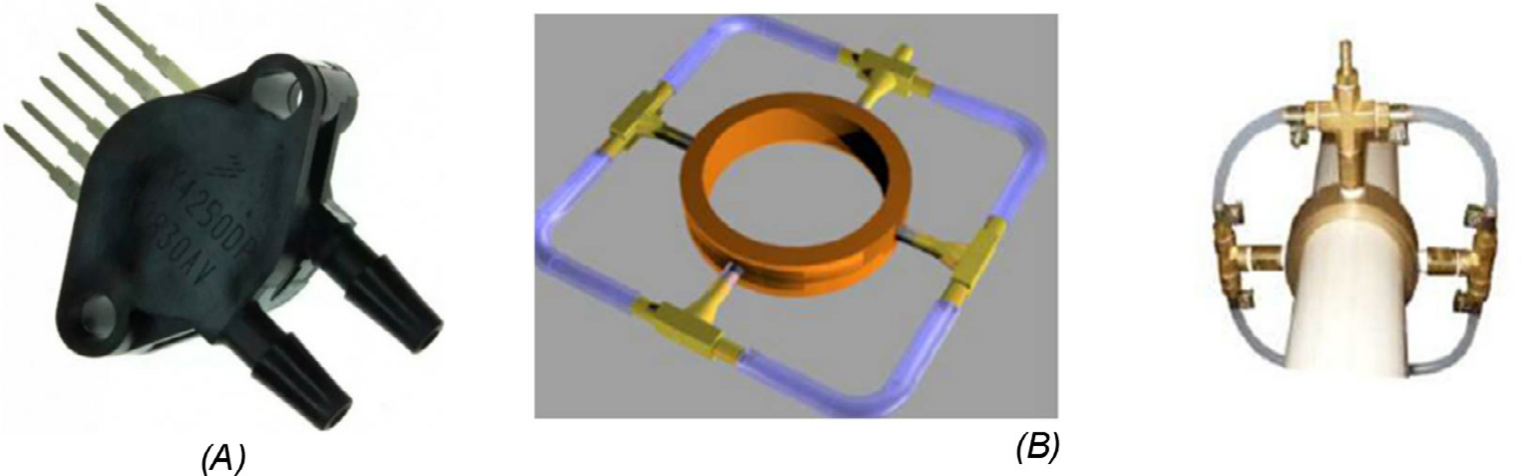
Detail of the pressure transducer used (A), and the model of the integral pressure sensors (B).

**Table 2:** Technical specifications of the pressure transducers model MPX5700DP.

Specification	Value
Pressure range (kPa)	15–700
Supply voltage (V)	4.75–5.25
Supply current (mA)	Máx 10
Accuracy (%)	±2.5
Response time (ms)	1
Operating temperature (°C)	−40 to 125
Transfer function	$P\left(\text{kPa}\right)=155.54\;V_{\text{out}}\left(V\right)-31.11$
- Data acquisition system: The eletromagnetic flow rate sensor and pressure transducers were connected to a data acquisition system model PCI 6221 (National Instruments Brazil, São Paulo, Brazil), managed by a computer interface developed using the LabVIEW software (National Instruments Brazil, São Paulo, Brazil) installed on a microcomputer (Fig. 5). Table 3 shows the technical specifications of the data acquisition system model used in experiment.

**Fig 5:**
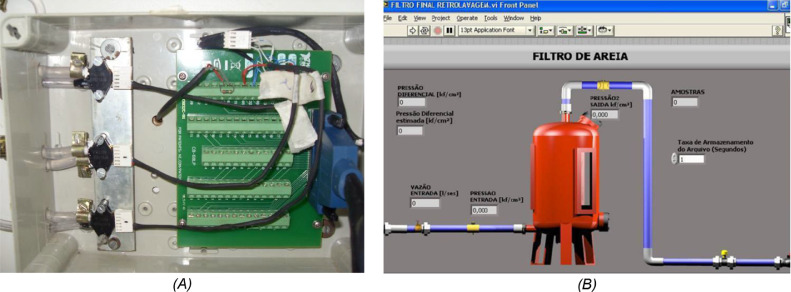
Detail of the pressure transducer with the data acquisition system installed on a microcomputer (A), and the interaction interface developed in the LabVIEW software (B).

**Table 3:** Technical specifications of the data acquisition system used.

Specification	Value
Number of input channels	16
Number of output channels	4
Sampling rate (KS s^−1^)	250
Resolution (bits)	16
Minimum voltage range (mV)	−200 to 200
Maximum voltage range (V)	−10 to 10
- Pump: it was used a centrifugal pump with nominal flow of 100 m^3^ h^−1^ (Meganorm Bloc, KSB, rotor diameter of 332 mm, São Paulo, Brazil) with an three-phase electric motor of 25 cv, 1760 rpm, and 60 Hz.
- Water tank: the water used in the experiment was allocated in a tank of 50 m^3^. In order not to modify the hydraulic characteristics of the equipment in the process due to the clogging of the filter media, the water used in the experiment originated from the municipal supply system of Campinas in the state of São Paulo (SP), Brazil.

**Fig 6:**
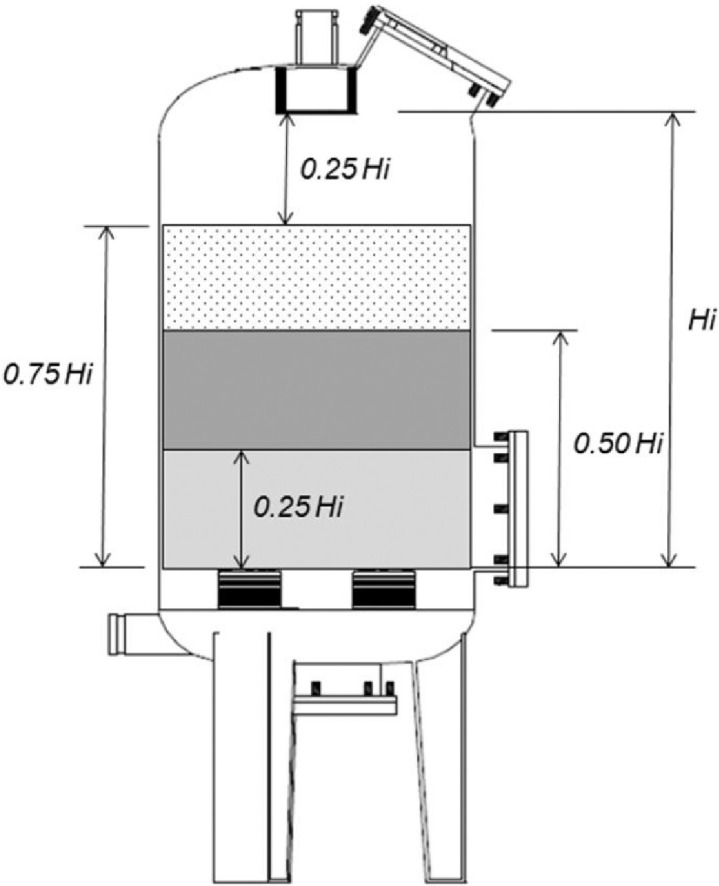
Illustration of the methodology for defining the filter bed heights to be evaluated in the sand filter backwash process.

The filter medium used was silica sand, with three particle sizes, designated G1 (particle size range of 0.5–1.0 mm, with a uniformity coefficient – CU = 1.50), G2 (range of 0.8–1.2 mm with CU = 1.27) and G3 (range of 1.0–1.5 mm with CU = 1.34). The determination of the sand particle size distribution was based on the ABNT EB-2097 standard (1990) [9]. These sand particle sizes were combined with three different filter bed heights (H1, H2 and H3). The heights were defined as equal fractions of the useful height of each filter model to fluidise (Hi) (Table 1), with a limited maximum height to enable a minimum expansion of interest. In the experiment the minimum expansion was 25% of the height of the static layer, according Brouckaert (2004) [10]. Fig. 6 illustrates the methodology for defining the filter bed heights.

The experimental procedure was based on directing the water inside the sand filter at a certain surface velocity (flow rate ratio for the cross-sectional area of the sand filter) in the backwashing direction (upward). Video 1 below shows a backwashing test on one of the evaluated sand filters in the experiment.

Video 1. Video of the backwashing process evaluation in one of the evaluated sand filters.

After the filter bed expansion, the expanded height was measured (visually), as well as the pressure loss (differential between the pressures at the inlet and outlet of the sand filter).

**Fig 7:**
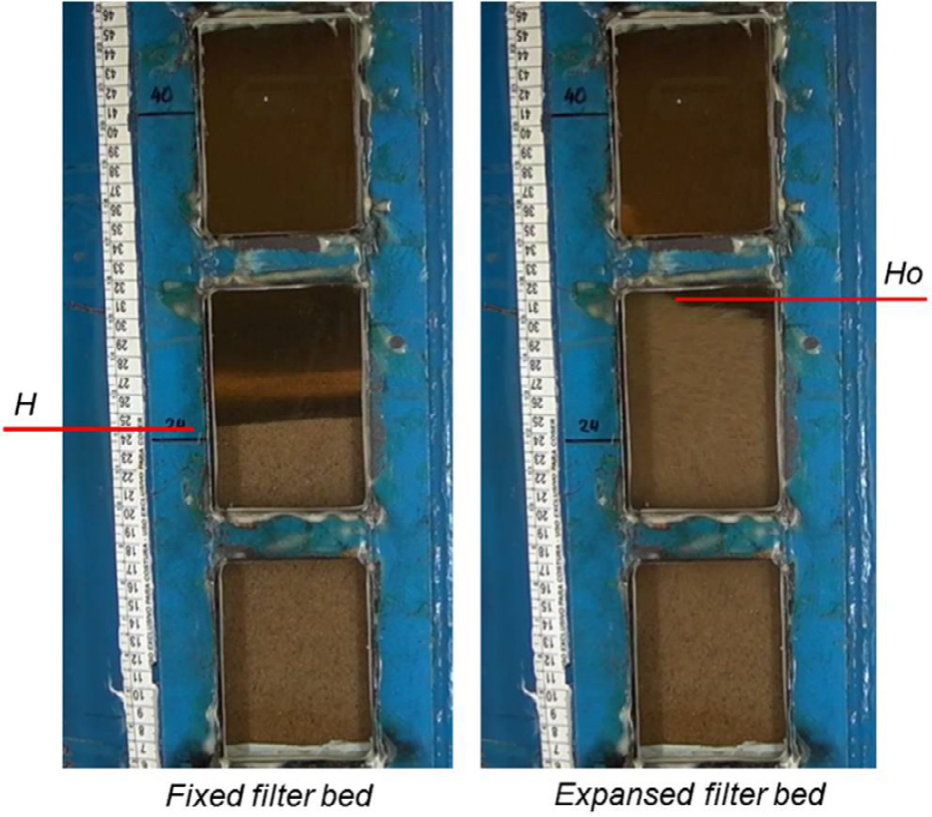
Example of the backwash process occurring inside the sand filters, with details of the expanded height measurements.

The surface velocities were defined based on extreme values, equally spaced above and below the reference value of 0.01 m s^−1^ proposed by Pizarro Cabello (1996) [11] for backwashing processes in sand filters used in micro irrigation. Fig. 7 illustrates the moment of reading the height of the expanded filter bed has occurred.

In projects that aim to use sand filters, it should be considered that equipment with a larger area must be supplied by a pump with greater power, given the need for greater flow for the same surface velocity. In the results presented by de Deus et al. (2020) [7], a limitation in surface velocity was observed in the backwash process evaluation of sand filters with different areas (maximum value of 0.07 m s^−1^ for filter F1, 0.045 m s^−1^ for F2, and 0.044 m s^−1^ for F3).

Initially, to evaluate the filter structure influence on pressure loss (ΔP), the backwash process was performed with the empty filter. 81 evaluations were performed with empty sand filter condition (three sand filters evaluated at nine surface velocity, in three repetitions).

The filters filled with filter media were also evaluated, determining the pressure loss (ΔP) as well as the height of expanded filter bed (Ho). With the Ho values it was possible to estimate the percentage of filter bed expansion (E) (Eq. (1)).

(1) $$E=\left(\frac{H_{0}-H}{H}\right)100$$

where E is the percentage of filter bed expansion (%), H_0_ is the height of expanded filter bed (mm), and H is the height of the static filter bed (H1, H2 and H3) (mm).

The relationship between surface velocity and the percentage of filter bed expansion was also determined for each experimental combination. In total there were 729 backwashing evaluations with filled sand filters (combination of three sand filters, three sand particle sizes, three filter bed heights, nine surface velocities, with each repeated three times).

Adapting the procedure described by Burt (2010) [12], the pressure loss caused by the filter media was evaluated as a function of the surface velocities evaluated. For this, a numerical difference was made between the sand filter pressure loss with filter media, and the pressure loss of the empty equipment, for the same surface velocity evaluated.

**Fig 8:**
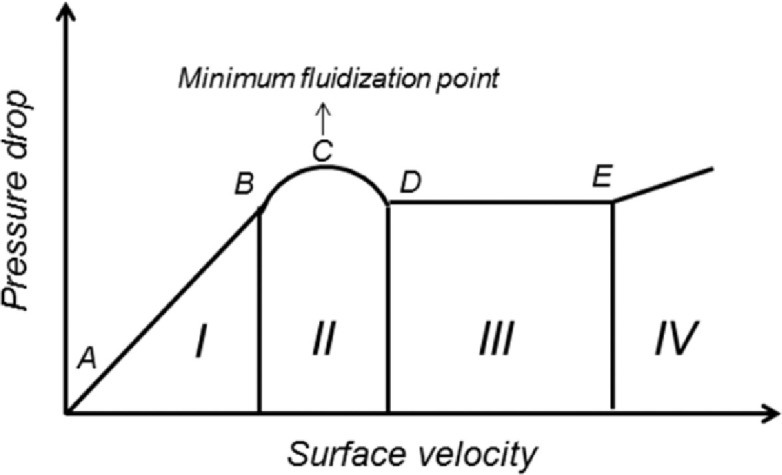
Illustration of the theoretical behavior of pressure loss in fluidized beds, being: I (AB) - static filter bed range, II (B) - bed expansion characterized by the equilibrium between pressure loss and particles weight, C - minimum fluidization point, III (DE) - most of the filter bed in fluidisation, IV - particle drag (adapted from Gupta and Sathiyamoorthy (1999) [4]).

This procedure aimed to evaluate a methodological proposal for indirect evaluation of the filter bed minimum fluidisation velocity for each experimental combination, which according Cleasby and Fan (1981) [13] is the point of intersection between the pressure loss curve of the static or non-expanding filter bed (curve range with linear increase of pressure loss with the superficial velocity) and fluidized filter bed (stabilization range pressure loss as a function of superficial velocity). For Gupta and Sathiyamoorthy (1999) [4] it is the point where the pressure loss and buoyancy are in equilibrium with the apparent weight of the particles, with little contact between them. According to some authors, the minimum fluidisation point is fundamental for the optimization of the sand filters backwashing process [13,14]. According Gupta and Sathiyamoorthy (1999) [4] when the fluid has a lower superficial velocity, the particle interstices generate enough resistance to cause a pressure loss, increasing with the superficial velocity increase. However, as the particles start to separate from each other (filter bed expansion), the pressure loss becomes constant and equal to the particles weight, regardless of the surface velocity increase. Fig. 8 illustrates the theoretical behavior of pressure loss in fluidized beds.

To verify the reliability of the minimum fluidization velocity methodology, an experimental determination was made for each experimental combination.

The duration of each test was 180 s, from which a stabilised range of 100 s was used to record the information. The recording rate was adjusted by the data acquisition system but was had an average of one data point per second.

## Appendix Supplementary materials

Image, application 1

Image, video 2
