# Supplementary material for: Methodology for hydraulic characterisation of the sand filter backwashing processes used in micro irrigation
Source: MethodsX. 2020 Jun 13;7:100962. doi: 10.1016/j.mex.2020.100962 (PMC7327839; doi:10.1016/j.mex.2020.100962)
Supplement: Supplementary file 1 [file mmc1.docx]

**DATA STATEMENT**

I am not specifying any data sets. The data will still be used to generate new information.
